# Comparing Metacognitive Representations of Bodily and External Agency

**DOI:** 10.1523/ENEURO.0164-25.2025

**Published:** 2026-01-02

**Authors:** Angeliki Charalampaki, Heiko Maurer, Lisa K. Maurer, Hermann Müller, Elisa Filevich

**Affiliations:** ^1^Bernstein Center for Computational Neuroscience Berlin, Berlin 10115, Germany; ^2^Berlin School of Mind and Brain, Humboldt-Universität zu Berlin, Berlin 10115, Germany; ^3^Department of Psychology, Faculty of Life Sciences, Humboldt-Universität zu Berlin, Berlin 10099, Germany; ^4^Neuromotor Behavior Laboratory, Justus Liebig University, Giessen 35394, Germany; ^5^Institute of Sport Science, Justus Liebig University, Giessen 35394, Germany; ^6^Center for Mind, Brain and Behavior of the Universities Marburg, Giessen and Darmstadt 35032, Germany; ^7^Hector Institute for Education Sciences & Psychology, University of Tübingen, Tübingen 72072, Germany

**Keywords:** EEG, metacognition, motor control, movement, outcome, sense of agency

## Abstract

We studied the role of movement and outcome information in forming metacognitive representations of agency. Human participants (*N* = 40; 25 female, 15 male, 0 diverse) completed a goal-oriented task: a semivirtual version of a ball-throwing game. In two conditions, we manipulated either the visual representation of the throwing movement or its proximal outcome (the resulting ball trajectory). We measured participants’ accuracy in a discrimination agency task, as well as confidence in their responses and tested for differences in the electrophysiological (EEG) signal using mass linear mixed-effect modeling. We found no mean differences between participants’ metacognitive efficiency between conditions. However, through exploratory analyses, we found that metacognitive sensitivity did not correlate between the two conditions and that the EEG signal differed between the two conditions during the agency discrimination task. We cautiously interpret these results as suggesting that although both movement and outcome information contribute to participants’ sense of agency, they may do so through distinct processes. These findings highlight the need for further research examining the potential neurophysiological differences corresponding to the conceptual distinction between bodily and external agency.

## Significance Statement

The sense of agency refers to the feeling of intentionally moving our body and, through that movement, affecting our surroundings. Empirical studies often explore this concept by focusing on either the bodily representations (how participants move their bodies) or the external representations (how this movement affects the environment) of voluntarily performed actions. In other words, these two types of representations are often assumed to be equivalent. This work challenges that assumption using a goal-oriented motor task, electroencephalographic data, and bias-free methods from metacognitive literature.

## Introduction

The subjective experience that we are the authors of our intentional actions is referred to as the sense of agency. This is an umbrella term that can refer to many, potentially distinct, cognitive processes. In fact, previous work has pointed at this breadth of the scope in the term and aimed to distinguish different operational definitions of agency (Pacherie, 2007; Synofzik et al., 2008; Gallagher, 2012; Christensen and Grünbaum, 2017, 2018; Wen, 2019). In particular, there is an ongoing discussion on the similarities and differences between bodily and external operational definitions of agency (Kalckert and Ehrsson, 2012; Christensen and Grünbaum, 2017; Wen, 2019). The difference lies in whether the definition used in a study of agency over an action (“I threw the ball in the basket”) is strictly limited by the boundaries of our body (the movement: “I moved my arm to throw”) or the definition is broad enough to also include agency over the consequences of our actions that go beyond the boundaries of our body (proximal outcome: “The ball flight trajectory” or the distal outcome: “The ball landed in the basket”). However, in the literature, this dichotomy in the operational definition often goes unnoticed, despite its importance in designing experimental protocols and models that adequately address this concept. These two definitions are not only conceptually distinct but differ also in their neural correlates. A review of fMRI studies on agency found a distinct activation pattern based on whether the experimental protocol employed focused on either the bodily or external agency (Charalampaki et al., 2022), emphasizing the broader implications of this dichotomy and the necessity to study these two aspects of agency in relation to one another, to explore where they converge or diverge both at the behavioral and at the neuronal level. In what follows, we will first discuss the most common model used to explain the human sense of agency: the comparator model (Frith et al., 2000), which is often used interchangeably to describe both bodily and external sense of agency. There is evidence that both agency over the movement and agency over the outcome are reduced by similar types of experimental manipulations; but there are also conflicting results on their relative importance in our sense of agency, which challenges the notion that they can be used interchangeably. We will then motivate our study by outlining how borrowing methods from the metacognitive literature can inform this discussion. Finally, we will offer a brief literature-based overview of the EEG signatures of agency and describe how this motivated our exploratory analysis.

According to the comparator model, whenever we make an intentional action, a copy of the motor (efferent) commands issued to make the necessary movements is used to make predictions about the consequences of the movement. These predictions are then compared with the actual afferent sensory feedback (associated with our moving limbs). When these two match, we experience authorship of the movement; when they do not, we experience a loss of agency. While the comparator model was originally used to explain agency over our bodily movements (Frith et al., 2000), it is often extended to explain the agency we experience over the external consequences accompanying our actions (Haggard, 2017). That is, it is usually assumed that the comparator can also make comparisons between predictions and feedback for the sensory consequences that go beyond our bodies. However, this assumption has been challenged. In particular, because current experimental designs have either failed to or provided mixed results regarding the involvement of efference copies in predicting the sensory consequences of outcomes beyond the body (Christensen and Grünbaum, 2018; Dogge et al., 2019). Further, participants can experience agency even for actions carried out by another person (Wegner et al., 2004; Tieri et al., 2015). Meaning that agency can be experienced in the absence of an efference signal (but see Kilteni et al., 2021 for the importance of efference copies in the sensory attenuation). Therefore, it is important to question the suitability of the comparator model to explain the sense of agency over external consequences of movement.

On the one hand, one series of studies shows that subjective reports on participants’ sense of agency are affected by manipulations of the visual representations of both a movement and its outcome. In these studies, participants reported experiencing a diminished sense of agency over an action when they fail to achieve a specific goal (van der Weiden et al., 2013), when the outcome was different than their predictions (Sato and Yasuda, 2005), or when the predicted outcome was delayed (Spengler et al., 2009; Farrer et al., 2013). Similarly, participants reported a lower sense of agency over a movement they made when the visual feedback of the movement they performed was manipulated spatially (van den Bos and Jeannerod, 2002; Farrer et al., 2003; Padrao et al., 2016) or temporally (Farrer et al., 2003; Krugwasser et al., 2019).

On the other hand, another line of work has aimed at directly comparing the relative precision of agency representations based on movement and outcome prediction violations, with conflicting results. While some studies found that participants were more sensitive to violations of outcome predictions as compared with violations of bodily predictions, other studies have found the opposite. For example, David et al. (2016) introduced a sensorimotor mismatch by temporally delaying the visual feedback, either at the point of movement initiation or at the onset of the movement outcome. They found that participants’ agency ratings decreased most sharply when the timing of the outcome was manipulated. Similarly, Wen et al. (2015) asked participants to click on a keyboard to control the position of a moving dot, aiming to make rapid movements to reach a visual target. The visual feedback was manipulated by introducing a temporal lag, and the level of control on the movement was manipulated by ignoring some of the participants’ commands. The latter manipulation was meant to introduce a sensorimotor mismatch so as to affect whether participants reached their goal, sometimes improving their success rate. Participants’ agency reports reflected whether they succeeded with their goal despite some of their commands being ignored, albeit only under some conditions, where the delay introduced was long. Finally, Oishi et al. (2018) extended these findings by showing that reaching a goal can retrospectively influence participants’ agency: Participants moved a cursor toward a changing color target (on some trials, the cursor moved with a delay). However, independently of the delay introduced on the movement, reaching the intended outcome (successfully reaching the target while it was on a specific color, as opposed to it being of a different color) was sufficient for participants to report feeling stronger agency.

The studies summarized above point toward outcome prediction violations overriding movement prediction ones, suggesting in turn that the representation of outcome predictions might affect participants’ agency more strongly than representations of movement predictions. But, as mentioned above, other studies directly contradict this conclusion, by showing that participants are more sensitive to violations of motor predictions as compared with violations of outcome predictions. For example, Metcalfe and Greene (2007) asked participants to move a cursor toward downward-moving visual targets and, on some trials, manipulated the movement feedback by introducing “turbulence” on the cursor's movement. They also manipulated the outcome by introducing false outcome hits (viz., the target appeared as being “magically” touched). Without turbulence, participants’ ratings of agency (or control) matched the proportion of targets they hit. In other words, in the absence of movement prediction violations, the more targets they hit, the higher their control rating. However, when there were either movement manipulations or outcome manipulations, participants’ control ratings more closely followed the relation between their motor act and the visual feedback of it, irrespective of how many targets it appeared as they had touched. In a similar study, Metcalfe et al. (2013) extended their findings by manipulating both the motor aspect of the action (turbulence) and the probability of a target being exploded upon touch. They showed that while both types of violations negatively affected participants’ agency, violations of the movement predictions had a more notable effect on participants’ ratings of control, and this was independent of participants’ performance, measured as the number of targets participants hit.

As a whole, this body of literature demonstrates that both bodily and external prediction violations, probed with manipulations of the feedback on either the movement or its outcome, can affect explicit reports. Importantly however, the different results also emphasize the importance—and challenges—of differentiating between different experimental manipulations in the study of agency. Beyond the conceptual differences driven by different experimental operationalizations of the sense of agency, there are two methodological aspects that limit the interpretability of the findings: All of the experiments summarized above measure participants’ sense of agency using subjective reports, which are prone to biases and this could explain the apparent diversity of the findings regarding the relative importance of movement and outcome predictions in our subjective experience of agency. Also, some of the studies compared participants’ sensitivity in detecting prediction violations of movement and outcome using manipulations that differ substantially between the two conditions, for example, manipulating the movement feedback with turbulence versus manipulating the outcome feedback by controlling the probability of an outcome occurring (Metcalfe et al., 2013). This challenges the comparison of the precision of these two sources of information as we do not know how these two different levels of action description relate to one another. To deal with these complications, while studying agency with explicit reports, one can instead measure the precision of explicit representations of agency using tools borrowed from research in metacognition (Fleming and Lau, 2014). That is, one can measure metacognitive representations of the agency judgments that lie on one representational level above, allowing for a comparison of their relative precision. This is the approach we chose.

In addition to behavioral data, we chose to collect EEG data to investigate possible neuronal-level differences arising from violations of bodily and external prediction linked with the sense of agency during action execution. Currently there is limited literature regarding electrophysiological markers of agency. Existing studies have primarily examined changes in the EEG signal amplitudes. In other words, they have examined the positive and negative deflections of the EEG signal (event-related potentials, ERPs) either during (Padrao et al., 2016; Han et al., 2021; Ren et al., 2023; Gomez-Andres et al., 2024) or following action execution (Kühn et al., 2011; Bednark and Franz, 2014; Vastano et al., 2020). However, these studies differ from one another significantly both in the experimental designs and in the ways agency was measured. Some involved Eriksen Flanker stimuli followed by hand movements without behavioral measures of agency (Padrao et al., 2016; Gomez-Andres et al., 2024); others required pressing a button to prevent a sound, measuring agency implicitly through auditory sensory attenuation reflected in EEG (Han et al., 2021). Additional variations include using a go/no-go task with feedback on performance (Ren et al., 2023), button presses (Sidarus et al., 2017), cursor movements toward targets (Bednark and Franz, 2014) with explicit agency attribution judgments, or button presses followed by an auditory stimulus, where agency was measured with explicit self-other ratings (Kühn et al., 2011) or implicit measures of agency (Vastano et al., 2020). Given these methodological differences, it is unsurprising that ERP components linked to agency differ substantially in their temporal and spatial characteristics. Negative deflections observed include the frontal event-related negativity occurring ∼100 ms after erroneous actions (Padrao et al., 2016; Gomez-Andres et al., 2024) and N400 components over parietal areas in response to externally generated errors (Padrao et al., 2016). Positive deflections have been identified as well, such as the P3 component over frontocentral regions, which was modulated by self-other agency judgments (Kühn et al., 2011) or varied with levels of agency under ambiguous action–outcome coupling (Bednark and Franz, 2014), and a P600 component showing differences between self and externally generated errors (Gomez-Andres et al., 2024).

Other studies have focused on the EEG frequency content and power changes across frequency bands, but these results remain inconsistent. Some evidence points toward increased alpha and beta oscillation power correlating with increased agency (Zito et al., 2023), while others report decreased alpha power with increased agency (Kang et al., 2015; Wen et al., 2017). Additionally, reduced frontal alpha-band connectivity has been associated with increased action control (Kang et al., 2015). Beta oscillation phase coherence changes have shown modulation based on control level across widespread regions, whereas gamma oscillation phase coherence varied with agency level over central and left parietal areas (Kang et al., 2015). It has also been proposed that coupling of gamma-band oscillations between parietal and presupplementary motor areas relates to positive agency (Ritterband-Rosenbaum et al., 2014). Lastly, findings regarding theta oscillations and their role in agency perception remains similarly complex. Enhanced theta power over left frontocentral and parietal electrodes has been linked to positive, and over temporoparietal electrodes to negative agency (Jeunet et al., 2018), whereas decreased theta synchronization over centrotemporal regions has been associated with increased agency (Zito et al., 2023). In the absence of widely accepted neural markers of sense of agency, we performed a series of exploratory analysis on EEG signals to identify differences associated with variations in the salience of agency experience.

**Figure 1.**
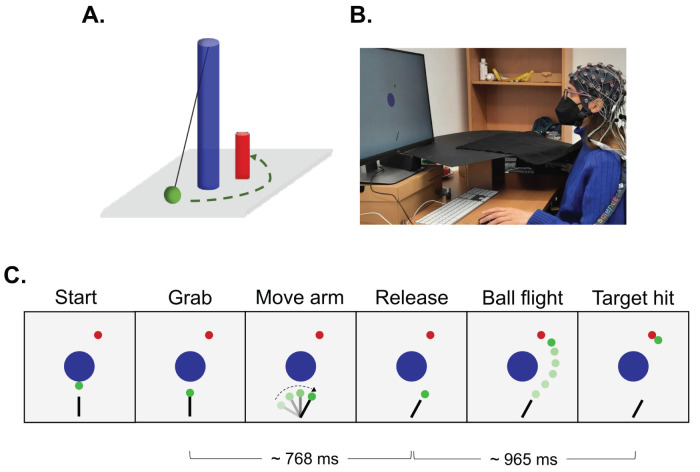
***A***, The Skittles game. The aim of the game is to swing the ball (green) attached to the pole (blue) with a rope to hit the target (red). ***B***, Experimental setup. Participants sat in front of the screen, placed their right hand on the custom-made lever (which was hidden below an occluder), and used a computer mouse with their left hand to register their responses. We recorded the EEG signals from 62 active electrodes placed on an elastic head cap. ***C***, Virtual version of the Skittles game. For each action, the ball, lever, and target appeared simultaneously on the screen. Participants could then grab the ball to start their action. On average, for each action, participants released the ball 765 ms after grabbing it, and the ball traveled for a mean of 965 ms from the point of release to the target. From the moment participants released the ball, the ball disappeared from the screen for ∼202 ms (*M* = 202.36 ± 0.21) to avoid participants being able to identify the manipulated interval based on the jump in the position of the ball due to the manipulation.

In this study, participants made movements in a deterministic, goal-oriented motor task in which we could tightly manipulate both the visual representations of the movement and the outcome. The motor task was a semivirtual version of the “Skittles” game (Müller and Sternad, 2004; Fig. 1). In the Skittles game, in which a ball is attached to a pole with a rope, the aim is to grab and throw the ball in such a way to hit the target placed behind the pole. In this task, we could manipulate both the visual representations of the movement and the outcome. On each trial, participants completed a motor action (grab and throw the ball to hit the target) twice in succession; they selected which of the two actions they felt were more in control (only one matched their action) and then rated their confidence in that decision. We hypothesized that representations of movement and outcome differentially affect participants’ metacognitive representations of their sense of agency and that this would also be reflected as behavioral differences. We also explored the data and sought for any potential differences at the neural level based on the underlying electroencephalography (EEG) data. Specifically, we focused on the FCz electrode, as prior evidence suggests that centro-frontal regions are involved in online monitoring of action execution, outcome processing, and other agency-related processes, such as the attenuation of the sensory consequences associated with voluntary actions. Given the exploratory nature of this analysis and the precise timing of the experimental design, we examined time-locked differences in EEG signal amplitude.

## Materials and Methods

We assessed whether violations of visual representations of a movement and its consequences on the environment equivalently affect participants’ sense of agency using behavioral and electroencephalography data (EEG). The study was preregistered (https://osf.io/SJURK), and we report any deviations from the preregistered plan. The ethics committee of the Institute of Psychology of the Humboldt-Universität zu Berlin approved the experimental procedures (2017-17), which were in line with the Declaration of Helsinki. Human subjects were recruited at a location which will be identified if the article is published.

### Participants

Our preregistered plan was to collect 40 clean datasets from participants who passed the inclusion criteria: no neurological or psychiatric history, normal or corrected-to-normal vision, right-handedness, and proficiency in English. The sample size was determined based on a previous study in which the Skittles task was embedded in a motor metacognitive experimental design (Arbuzova et al., 2021). To that extent, we collected data from 52 participants. We only included participants who correctly selected the interval in which they were in control (the online feedback matched their action), 60% or more of the total number of trials belonging to each condition. Therefore, we excluded five participants with very low discrimination accuracy (<60%) in at least one of the two conditions. In deviating from our preregistered plan, we further excluded the datasets from seven participants with especially variable discrimination performance throughout the experimental session. We deemed performance to be variable for these participants based on a visual inspection of the adaptively changed manipulation magnitude of the visual feedback (see the description of the staircasing procedure below) prior to analyzing the data. This resulted in a final sample of 40 participants (mean age = 26.60 years ± 5.60, 25 female, 15 male, 0 diverse, mean handedness score = 90.60 ± 14.50; Oldfield, 1971). Due to technical issues, the EEG signal from two participants was not recorded, but we nevertheless used these participants’ data in the behavioral analysis. Participants signed a written informed consent and for their time, were compensated with €8/h or course credits.

### Apparatus and stimuli

For the motor task, we adapted the virtual version of the “Skittles” game, first described by Müller and Sternad (2004). In the Skittles game, a ball is attached to a pole with a rope, and a target—the Skittle—is placed behind the pole (Fig. 1*A*). The goal is to hit the target by swinging the ball around the pole. In the virtual version of the Skittles game, participants had a top-down view of the scene: The central pole was positioned in the middle of the scene [blue circle, position (*x*,*y*) = (0,0) m, with a radius = 0.25 m]; the ball initially appeared hanging next to the pole (green circle, radius = 0.05 m); and the target was, when viewed from above, top-right of the pole [red circle, position (*x*,*y*) = (0.20,0.70) m, radius = 0.05]. The massless rope (spring constant *k* = 1 N/m) was not visible on the scene but was taken into consideration when estimating the ball trajectory. Participants grabbed, moved their forearm, and released the ball using a custom-made lever. The lever rotated on the end nearest to the participants, so that they could swing their arm horizontally around the elbow point. The angle of their hand was recorded online with the aid of a goniometer (Novotechnik, RFC4800 Model 600, 12 bit resolution, 0.1° precision), and all the data from the lever were recorded with a sampling rate of 1 kHz using a LabJack T7 data acquisition device (LabJack). This allowed us to depict the virtual lever in the lower middle part of the scene [(*x*,*y*) = (0, −1.5) m] as a black rectangular (0.4 m length, 0.04 m width) that pivoted along with the physical lever. Participants grabbed the virtual ball by placing their index finger on the touch switch on the tip of the lever and released it by lifting their finger. The Skittles game is deterministic. That is, the ball trajectory can be determined at the point of ball release by the angle of the lever and the velocity of its tip (Müller and Sternad, 2004; Sternad et al., 2011). Therefore, we could display the ball trajectory in real time with a maximum possible flight duration of 2 s. Once participants lifted their finger, the ball disappeared for ∼200 ms (*M* = 202.36 ± 0.21) to avoid a jump in the ball position when the ball trajectory was manipulated (see below). Each motor task started with only the central pole displayed, and with a pseudorandomized delay (0.40–1.00 s), the lever, the ball, and the target appeared on the screen. We programmed the task in MATLAB (R2016b, MathWorks) and Psychtoolbox-3 (Brainard, 1997; Pelli, 1997; Kleiner et al., 2007). Throughout the experiment, participants had no visual access to either their arm or the real lever (an opaque piece of cloth was held above their arm, without interfering with the movement), so they had to rely on the visual feedback they received on an LCD monitor (2,560 × 1,440 pixels, 0.61 × 0.345 m, refresh rate 60 Hz) located ∼0.6 m in front of them (Fig. 1*B*).

### Procedure

**Figure 2.**
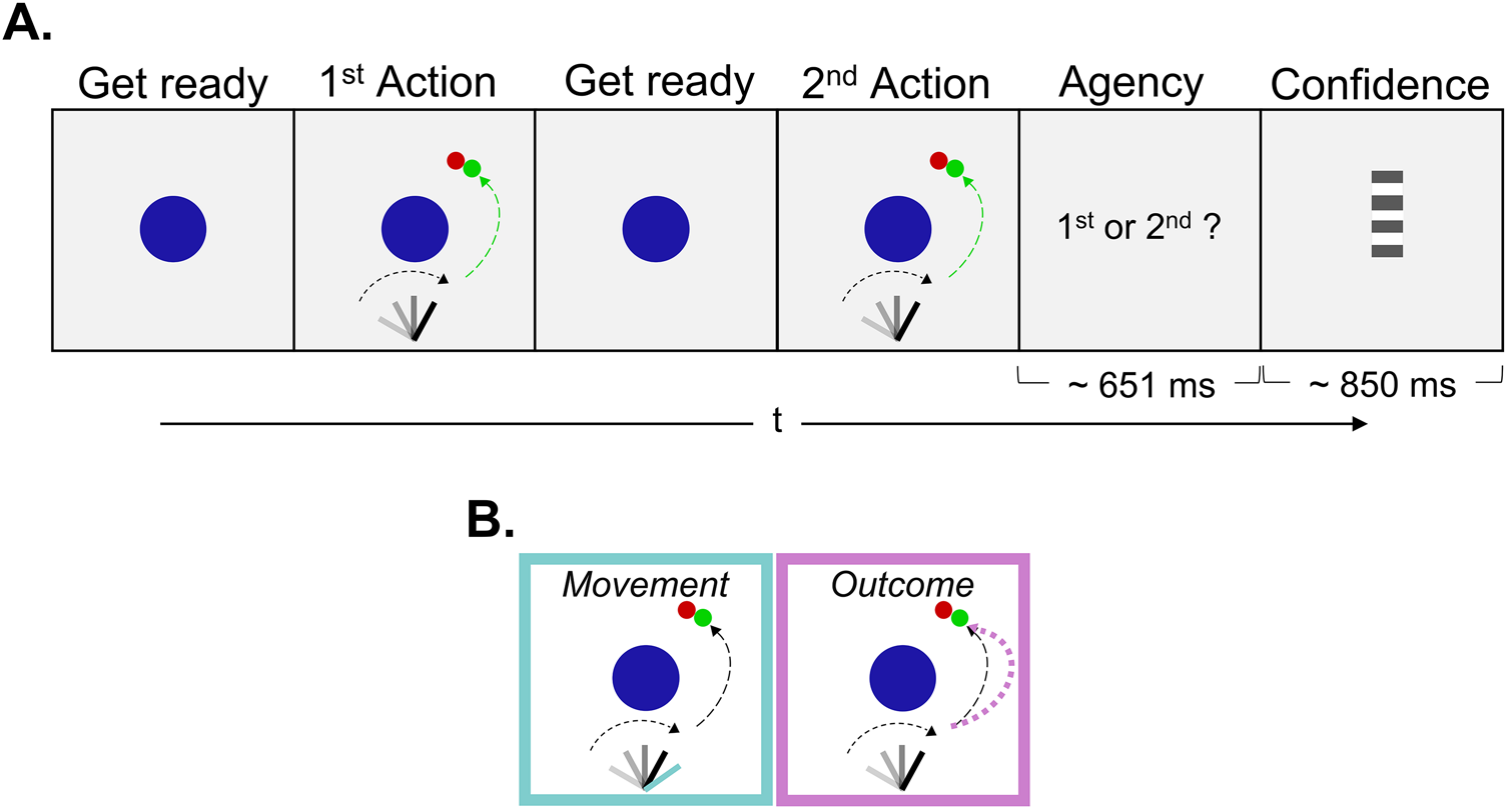
***A***, Experimental paradigm. In the main experiment, participants threw the ball twice in succession. The visual feedback accurately reflected their actions in only one of the two intervals. In the other interval, the feedback was manipulated based on the trial condition. At the end of the second action, participants selected which interval they felt more in control. Finally, participants rated how confident they were about their decision. ***B***, Experimental conditions. We manipulated the online visual feedback participants received on the screen in two conditions. In the Movement condition, we manipulated only the position of the virtual lever, which moved synchronously with the physical one but deviated a few degrees to the left or right (the black bar depicts the real position, the cyan bar shows the manipulated feedback participants receive). In the Outcome condition, we manipulated only the ball trajectory. The ball flew in an alternative trajectory (depicted with pink) with the same distal outcome (hit/miss the target) as the participants’ actual trajectory.

On each trial, participants performed the motor task two consecutive times (Fig. 2*A*). The online visual feedback they received matched their action in only one of the two intervals. In the other, the visual feedback was manipulated in one of two possible ways: either the position of the lever (Movement condition) or the trajectory of the ball (Outcome condition) did not match their own (Fig. 2*B*). Two previous studies used the semivirtual Skittles game and found that participants had accurate representations of both movement parameters—the velocity of the movement and the hand's angle position at the point of ball release—but also the ball flight trajectory and the distal outcome (Arbuzova et al., 2021; Charalampaki et al., 2023). We used a visuospatial manipulation to introduce violations of the predictions of the movement and proximal outcome for two reasons: first, because temporal delay could affect participants ability to monitor their action rather than simply introducing a mismatch between the prediction and the feedback of their action (for an extended discussion on the topic, see Wen, 2019; but also, Osumi et al., 2019) and second, to prevent participants from recognizing the visually delayed feedback as a delayed version of their action (Farrer et al., 2008). In the Movement condition, we only manipulated the angle of the lever (see details below, Online staircases). Therefore, the virtual lever always moved synchronously with the physical one but had a different angle, and the ball (which appeared after 202 ms; *M* = 202.36 ± 0.21) flew according to the participant's actual throw (based on the angle of participants’ real hand and velocity at the point of ball release). In the Outcome condition, the virtual lever precisely matched the participant's hand's movement. We manipulated only the ball trajectory, ensuring that it would always match the distal outcome of the participants’ real throw. Because on each trial (see below) participants compared two actions, we aimed to match the goal of each one participants’ action (Charalampaki et al., 2023). Therefore, we positioned the target slightly to the right of the pole to increase the chances of participants hitting it and emphasized in the instructions that they should always aim to hit it. At the end of the second action, participants discriminated in a two-interval forced choice task, in which of the two intervals they felt more in control (Fig. 2*A*). To do so, they could press the left or right computer mouse buttons with their left hand to report the first or second interval, respectively. Following the discrimination response, participants rated how confident they were that the interval they selected was the one in which they had complete control over the action seen on the screen, using a continuous virtual scale from “Very unsure” to “Very confident.” Participants moved up and down a computer mouse with their left hand, which controlled the position of a bar that appeared on a pseudorandom position over the virtual scale on each trial and then pressed either button on the mouse to register their confidence. During this point of the trial, participants could alternatively press the spacebar on a computer keyboard to mark the trial for exclusion (e.g., they pushed the wrong button in the discrimination step).

To familiarize participants with the virtual version of the task, participants first completed eight trials of the motor task in which the visual feedback always matched their action. After that, they did 40 training trials, half of which were Movement and half Outcome trials presented in pseudorandomized interleaved order. In these training trials, the trial ended after the discrimination response. Finally, participants completed 16 trials in which they also rated their confidence in their decision to become familiar with the steps of the main experiment. The main experiment consisted of 504 trials, split into 9 blocks, with breaks in between. Each block consisted of 56 pseudorandomized and interleaved Movement and Outcome condition trials.

The whole experiment, including EEG electrodes elastic cap preparation, took ∼3 h.

### Online staircases

To keep participants’ discrimination accuracy at ∼71% (Levitt, 1971), we used a separate online staircase (2-down, 1-up) for each condition. Both staircases operated on the velocity manipulation (|Δ*v*|). That is, in both cases, on each trial we added or subtracted the staircased |Δ*v*| to the actual lever velocity. We then displayed, on each time point, the lever position that corresponded to the manipulated velocity (on Movement trials) or the ball position given the manipulated release velocity (on Outcome trials). Specifically, on Movement trials we manipulated the angle of the lever by multiplying the |Δ*v*| by the time difference Δ*t* between two screen refresh frames in order to obtain the corresponding lever angle difference. On Outcome trials we manipulated the trajectory of the ball by changing the velocity of the real movement in |Δ*v*|, in order to create a manipulated ball flight trajectory. We pseudorandomized a parameter to determine whether we added or subtracted |Δ*v*|, in order to present a manipulated trajectory to the right or left of the real one, respectively. We deviated from this process if the alternative ball trajectory ended in a throw with a distal outcome (hit/miss the target) that did not match participants’ throw. In these instances, we first multiplied the |Δ*v*| with the opposite sign, and if this again led to an outcome incongruent from the real one, we used the nearest possible value that would. If none of these steps worked, we used the original staircase value and marked the trial for exclusion.

Importantly, on Movement trials, the ball flight trajectory in both intervals corresponded to the trajectory determined by the angular velocity of the participant's hand at the point of ball release. In other words, on Movement trials, the ball flight trajectory was displayed intact in both intervals. On Outcome trials, the virtual lever's angle in both intervals corresponded to the participant's hand position. In other words, on Outcome trials the virtual lever's angle was intact in both intervals.

The starting value of both staircases was 1, the step size was 0.15 in the training session and 0.10 in the main experiment, and the minimum possible value of the staircase was 0.21 for the training and 0.01 in the main experiment. We used larger step sizes in the training staircases as a means to roughly approximate a good starting point for each participant's discrimination ability, which would then be more finely staircased during the experiment.

### Data analysis

#### Data exclusion

In keeping with our preregistered plan, we excluded from both the behavioral and EEG data those trials in which the discrimination reaction time was above 8 s (mean excluded trials ± SD: 1 ± 2), trials marked for exclusion by the participants (mean ± SD: 4 ± 6 trials), and those trials in which the intervals differed in terms of the distal outcome (mean ± SD of trials participants missed hitting the target in either of the intervals: 63 ± 55 and mean trials in which participants threw the ball toward the left of the pole in at least one interval: 5 ± 11). Overall, we included a mean of 405 ± 70 trials from each participant (Movement trials: 198 ± 38 and Outcome trials: 207 ± 33).

In addition to our preregistered exclusion criteria, we excluded the (behavioral and EEG) data from the first blocks from those participants for whom the staircases were especially variable at the start of the experiment. Specifically, we excluded the first block from eight participants, the first two blocks from four participants, and the first four blocks from two participants. Data exclusion took place prior to any further data analysis.

#### Behavioral data

##### Metacognitive representations of agency

This design allowed us to quantify how well people can inspect their agency judgments. That is, we could use an estimate of metacognitive efficiency (Mratio; Maniscalco and Lau, 2012), which reflects the relationship between participants’ discrimination accuracy and confidence rating. Mratio aims to quantify the proportion of information available for confidence ratings, relative to that available in the discrimination decisions. Thus, as a unit-free measure, it allows for the comparison between conditions without being contaminated by potential biases in subjective reports. Participants with high Mratio typically rate with high-confidence trials in which they correctly identified in which of two intervals they had more control of the perceived action and rate with low confidence those trials in which they instead identified the interval in which they had less control of the perceived action. In contrast, confidence ratings from participants with low Mratio typically cannot differentiate between the trials in which they selected the correct interval from those they did not.

To estimate the Mratio, we used the maximum likelihood estimation method as implemented in the “*metaSDT*” R package (Craddock, 2021). For this analysis, we had to discretize the continuous confidence ratings, and we chose to do so using six equidistant bins. Before discretizing the ratings, we normalized them per participant (by subtracting their minimum confidence rating and dividing by the difference between their maximum and minimum confidence values, like Charalampaki et al., 2023), to account for possible differences in the range of confidence ratings used by each participant. Further, in line with the recommendations, to avoid having empty bins that would hinder the fitting procedure (Maniscalco and Lau, 2012), when fitting the models for each participant, we added 1/(2*number of bins) = 1/12 to each input vector containing the count of each confidence rating bin type.

We used the “BayesFactor” package (Morey and Rouder, 2018) to obtain the Bayes factors in favor of H1 (BF_10_ values) and the “rstatix” package (Kassambara, 2023) to estimate effect sizes and ran parametric correlations using the “ggstatsplot” package (Patil, 2021), all implemented in R (version 4.2.2, R Core Team, 2022). Prior to the statistical analyses, we log-transformed *d’* and scaled the confidence ratings (by mean-centering to the condition-specific mean, and normalizing by the condition-specific SD) because they were not normally distributed. We report mean values and standard deviations (*M* = mean value ± SD).

#### Electrophysiology recordings

We recorded electrophysiology (EEG) data using 62 active electrodes fitted on an elastic cap following the 10–20 system g.tec position montage (g.tec medical engineering). We placed two reference electrodes (one on each earlobe) and three electrodes around the right eye (above, below, and outer canthus) to record horizontal and vertical eye movements. We used AFz as the ground electrode and the electrode on the right earlobe as the online reference. The signal was digitized at a sampling rate of 1,200 Hz, online bandpass filtered between 0.5 and 200 Hz, and notch filtered between 48 and 52 Hz to remove line interference. We monitored the electrodes’ impedances throughout the experiment to keep them below 10 KΩ.

#### EEG analysis

##### Preprocessing pipeline

We preprocessed the EEG signal using the EEGLAB toolbox (Delorme and Makeig, 2004) and custom scripts implemented in MATLAB (R2020a, MathWorks). In line with our preregistered plan, we rereferenced the EEG signal to the average signal of the earlobe electrodes, filtered the signal using separate finite high-pass (1 Hz passband edge), and used low-pass (100 Hz passband edge) Hamming windowed-sinc finite impulse response (FIR) filters, notch filtered using a filter with passband edges at 49 and 50 Hz, downsampled to 600 Hz, and run Independent Component Analysis (ICA; *runica*). We first flagged the components, using built-in EEGLAB functions, and then visually inspected them to reject those involving eye movements and muscle contraction (focusing on those with a probability of over 0.90 for belonging to the eye or muscle component category).

##### Epoch extraction

After downsampling the EEG data to 250 Hz, we segmented them into epochs time locked to two events of interest: discrimination agency response and confidence response.

##### Report cues

(A) Discrimination agency response

For the discrimination response, we segmented the data into epochs time locked from −150 to 500 ms relative to when participants received the cue to register their choice of interval in which they experienced being more in control of (i.e., the appearance on the screen of the agency question). We baseline corrected these epochs using a time window of −150 to 0 ms. We excluded the epochs time locked to the discrimination response cue from one participant because they were noisy.

(B) Confidence response

For the confidence response, we used epochs that started −150 ms prior and ended 500 ms after participants received the cue to report their confidence in the preceding discrimination response. We baseline corrected for the time window 150–0 ms relative to the confidence response cue.

After epoching the data to each time-locking event, we first automatically rejected noisy epochs using the function *pop_autorej* with a threshold of 70 µV for extremely large fluctuations (Mean number of rejected epochs time locked at: Cue for discrimination: *M* = 102.87 ± 41.36; Cue for confidence: *M* = 107.11 ± 42.10). We then visually inspected the output to manually reject any remaining noisy epochs (Discrimination cue epochs: *M* = 151.66 ± 55.39; Confidence cue epochs: *M* = 164.45 ± 56.10). Finally, we excluded a small set of epochs from those trials in which, due to a technical error, at least one of the triggers fired more than once, introducing uncertainty in the timing (Rejected epochs time locked at: Discrimination cue: *M* = 1.50 ± 1.46; no epochs were removed time locked to the confidence cue). Therefore, for the final analyses we used the following number of epochs at each time-locking event: Discrimination cue epochs: *M* = 234.32 ± 70.53, min = 79; Confidence cue epochs: *M* = 224.97 ± 65.46, min = 76. We chose to focus our analysis on the FCz electrode, because ERP studies suggest that neuronal correlates of agency, be that online monitoring of movement execution, outcome feedback, and both implicit and explicit agency measures can be identified in this area (Kühn et al., 2011; Bednark and Franz, 2014; Padrao et al., 2016; Vastano et al., 2020; Han et al., 2021; Ren et al., 2023; Gomez-Andres et al., 2024) and scalp topography.

### Statistical analyses

To compare the amplitude of the EEG signal for the epochs created, we fitted linear mixed-effect models using the “afex” package (Singmann et al., 2023). To account for trial-level variability and maintain the within-subject structure of the data, we included all trials for each participant, without prior averaging. We then used cluster-based permutation tests to control for family-wise error rate (Maris and Oostenveld, 2007). For the latter, we permuted the factors’ labels (used on each model) on each trial and fitted linear mixed-effect models on each time point 5,000 times. For this purpose, we used sum-to-zero coding (*contr.sum*) to define the contrasts among the factors and their interactions. This approach centers each factor, allowing its effects to be interpreted relative to the grand mean rather than a reference level. For each main effect and interaction, we got clusters of neighboring time points within the epoch when there was a significant difference between the conditions (*p* < 0.05). Then, we estimated the sum of the *F* statistics for each cluster and saved the largest value. By repeating this process, we obtained the distribution of the sum statistics related to finding an effect by chance. We then tested whether the significant effects referred to time points that were both lower than our alpha (0.05) and fell above the 95th percentile of the time points of the sum-statistics distribution. These were exploratory analyses, in which we examined long epochs. Since the exact timing of the monitoring of the action and the experience of diminished agency is unknown, we prefer not to interpret the timing of the clusters but instead suggest that these exploratory analyses may reveal potential differences in the EEG signal.

For illustration purposes only, to explore how the main effects of these factors and their interactions across the scalp, we fit the data from the remaining 61 channels using the same linear mixed-effect models and then plotted the topographic maps of the mean beta estimates across the time points included in each of the significant clusters using the *eegUtils* package (Craddock, 2022) in R.

### Code accessibility

This study was preregistered (https://osf.io/SJURK), and any deviations from the preregistered plan are explicitly reported. The preprocessed data, as well as R and MATLAB scripts for the analyses, are freely available at (https://osf.io/sm7rg).

## Results

### Behavioral

**Figure 3.**
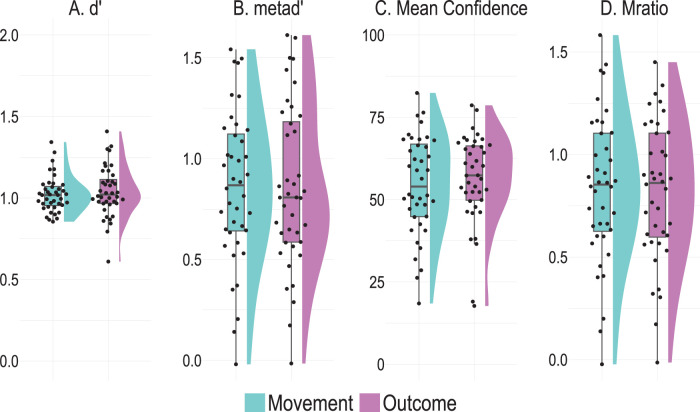
***A***, *d*’: Discrimination performance. ***B***, meta*d’*: Metacognitive sensitivity. ***C***, Mean Confidence ratings and ***D***, Mratio: Metacognitive efficiency. The violin plots depict the smoothed distributions of the data per condition, and the boxplots depict the interquartile range. The black circles represent the estimates per participant.

First, we compared participants’ discrimination performance—measured with *d*’—and found that there was no significant difference between the two conditions (*d*’_movement_ = 1.02 ± 0.11; *d*’_outcome_ = 1.04 ± 0.15; *t*_(39)_ = −0.42, *p* = 0.677, Cohen's *d* = −0.07, BF_10_ = 0.19; Fig. 3*A*). That is, the manipulation did not significantly affect the discriminability of the intervals in terms of control. This result confirms that the staircase procedure employed to keep participants’ accuracy stable in the discrimination task worked, allowing us to validly compare participants’ Mratio values between the two conditions. Further, we found that mean confidence ratings did not differ between the two conditions (Mean confidence_movement_ = 54.45 ± 15.03; Mean confidence_outcome_ = 56.31 ± 13.51; *t*_(39)_ = −1.71, *p* = 0.095, Cohen's *d* = −0.27, BF_10_ = 0.64; Fig. 3*B*). That is, we get weak evidence that participants’ confidence was comparable when they assess the accuracy of their representations of agency, both when the manipulation affected the visual feedback of their movement or the proximal outcome. Additionally, we found that participants’ metacognitive sensitivity, measured with meta*d’*, was similar between the two conditions (meta*d*’_movement_ = 0.86 ± 0.38; meta*d*’_outcome_ = 0.86 ± 0.40; *t*_(39)_ = 0.04, *p* = 0.968, Cohen's *d* = 0.006, BF_10_ = 0.17; Fig. 3*C*). Crucially, contrary to our preregistered hypothesis, we found that metacognitive efficiency, measured with Mratio, did not differ between the two conditions (Mratio_movement_ = 0.84 ± 0.36; Mratio_outcome_ = 0.82 ± 0.34; *t*_(39)_ = 0.25, *p* = 0.806, Cohen's *d* = 0.04, BF_10_ = 0.18; Fig. 3*D*). This shows that metacognitive representations of agency are equally sensitive to violations of predictions of the representations of the movement and outcome. These results held after removing two participants with negative Mratio. We found no significant difference in participants’ discrimination performance (*d*’) between the two conditions (*d*’_movement_ = 1.03 ± 0.02; *d*’_outcome_ = 1.04 ± 0.03; *t*_(37)_ = −0.23, *p* = 0.82, Cohen's *d* = −0.04, BF_10_ = 0.18). Further, there was no difference in participants’ confidence rating (Mean confidence_movement_ = 54.67 ± 2.40; Mean confidence_outcome_ = 56.21 ± 2.23; *t*_(37)_ = −1.47, *p* = 0.151, Cohen's *d* = −0.24, BF_10_ = 0.47) or metacognitive sensitivity (meta*d*’_movement_ = 0.90 ± 0.05; meta*d*’_outcome_ = 0.87 ± 0.06; *t*_(37)_ = 0.40, *p* = 0.694, Cohen's *d* = 0.06, BF_10_ = 0.19). Lastly, participants’ Mratio did not different between the two conditions (Mratio_movement_ = 0.88 ± 0.05; Mratio_outcome_ = 0.83 ± 0.05; *t*_(37)_ = 0.70, *p* = 0.497, Cohen's *d* = 0.11, BF_10_ = 0.22).

**Figure 4.**
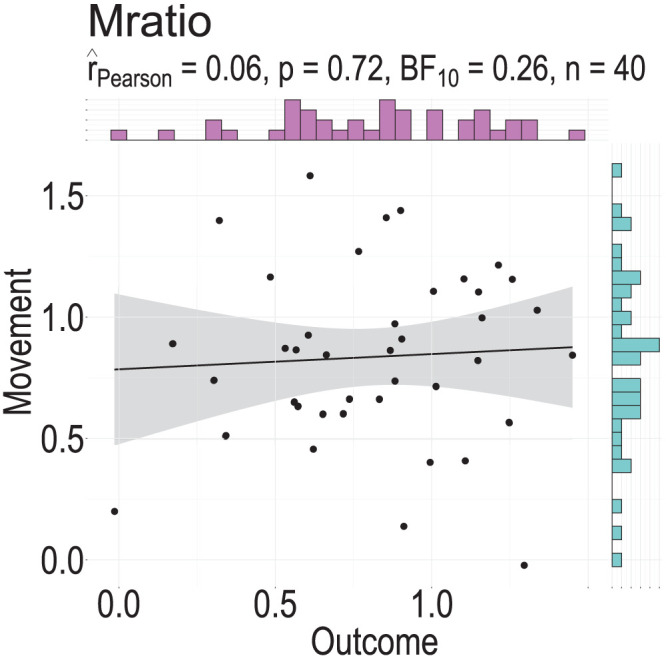
Correlation between the Mratios of the two conditions. No significant correlation was found between the Mratios of the Movement and Outcome condition. The black line represents the regression line, and the shaded area illustrates the 95% confidence interval. The black circles represent the Mratio for each participant and the histograms the distribution of the data for each condition (Extended Data Fig. 4-1 indicates that this pattern cannot be directly explained by differences in the magnitude of manipulation in each condition or by the confidence participants reported as a result).

In an exploratory analysis, we then examined the relationship between metacognitive representations of agency in the two conditions. We wanted to test whether participants’ sensitivity to movement and outcome prediction violations were not only comparable but also correlated. We found that the correlation was not significant (*t*_(38)_ = 0.36, Pearson's *r* = 0.06, *p* = 0.72, CI = [−0.26, 0.36], BF_10_ = 0.26, *n* = 40; Fig. 4). Excluding the two participants with negative Mratio from the correlation analysis had no effect on the results (*t*_(36)_ = 0.24, Pearson's *r* = 0.04, *p* = 0.81, CI = [−0.28, 0.35], BF_10_ = 0.25, *n* = 38). We interpret the absence of differences between the Mratios in the two conditions as evidence that visual manipulations of the movement and the outcome might similarly affect metacognitive representations of agency judgments at the population level, and the lack of correlation may point toward individual level variability. In other words, some participants are more sensitive to movement feedback manipulations compared with outcome feedback manipulations, while for others the opposite is true. Importantly, this individual level variability is not attributable to the staircase procedures employed to keep participants’ performance comparable across conditions. Stimulus variability was not associated with confidence ratings and did not account for the Mratio effects (see also Extended Data Fig. 4-1).

10.1523/ENEURO.0164-25.2025.f4-1 Figure 4-1 Velocity values and binned confidence ratings used per trial. Each sublot represents the values used per participant and condition (Movement upper, Outcome lower). As shown, confidence ratings are distributed across the full range of staircase values. Participants did not report high confidence exclusively on trials with larger velocity differences, nor low confidence only on trials with smaller values. Further, confidence ratings did not merely reflect the magnitude of the manipulation. This was shown by the absence of correlation between scaled confidence ratings (to normalize individual rating tendencies) and scaled stimulus variability (to account for differences in staircase characteristics) for each participant and condition. (Movement: t(38) = -0.41, Pearsons’s r = -0.07, p = 0.69, CI = [-0.37, 0.25], BF_10_ = 0.26, n = 40; Outcome: t(38) = -1.20, Pearsons’s r = -0.19, p = 0.24, CI = [-0.47, 0.13], BF_10_ = 0.46, n = 40) across participants and conditions. Finally, we found no condition-independent pattern that could explain the lack of correlation between the Mratios of the two conditions. To test this, we fitted two separate linear models predicting Mratio from staircase variability (model formula: Mratio ∼ scaled -SD), extracted the residuals (unexplained variability) from each model, and tested their correlation. The analysis provided moderate evidence against a correlation (t(38) = 0.36, Pearsons’s r = 0.06, p = 0.72, CI = [-0.26, 0.36], BF_10_ = 0.25, n = 40). Download Figure 4-1, TIF file.

### Electrophysiology

We then looked at the EEG signal to explore whether the different representation violations due to the movement or proximal outcome manipulations are linked with distinct brain signals.

#### Report cues

We then sought for differences in the EEG signal between conditions at the point participants were prompted to (A) report their discrimination decision and (B) their confidence rating. For readability, from now on, we will call them “Discrimination cue” and “Confidence cue” epochs, respectively. The aim of these analyses was to test for changes in the brain signals resulting from the different types of feedback participants received while performing the Skittles game. Specifically, we aimed to determine whether the brain signals recorded while participants were prompted to register their agency discrimination and confidence reports respectively differed significantly depending on whether the manipulation was introduced during the arm movement (Movement trials) or during the outcome phase (Outcome trials) of the action they had just completed. To compare for differences among the time points, we tested the following linear mixed-effect model EEG amplitude ∼ condition * confidence * accuracy in discrimination response + (1 | participant) applied to each time point of the Discrimination cue and Confidence cue epochs. For both, we used a time window starting at 0 s and finishing at 500 ms from when participants were prompted. These time windows were chosen based on the average time participants needed to respond (discrimination response: 651.36 ± 148.63 ms; confidence response: 844.10 ± 238.37 ms), to focus our analysis on the underlying signal linked with the discrimination and confidence task and avoid motor artifacts and other processes contaminate the neural signals. We expected that if the type of feedback plays a significant role in how participants experience their sense of agency during the action, then the neural signature would differ between conditions (Movement vs Outcome) during Discrimination cue and Confidence cue epochs. Such a result would align with the absence of correlation between the Mratios of the two conditions. It would also provide preliminary evidence that bodily and external agency are not simply facets of single, unified experience, and emphasize the need to study how each component contributes to an overall agency.

(A) Discrimination agency response

**Figure 5.**
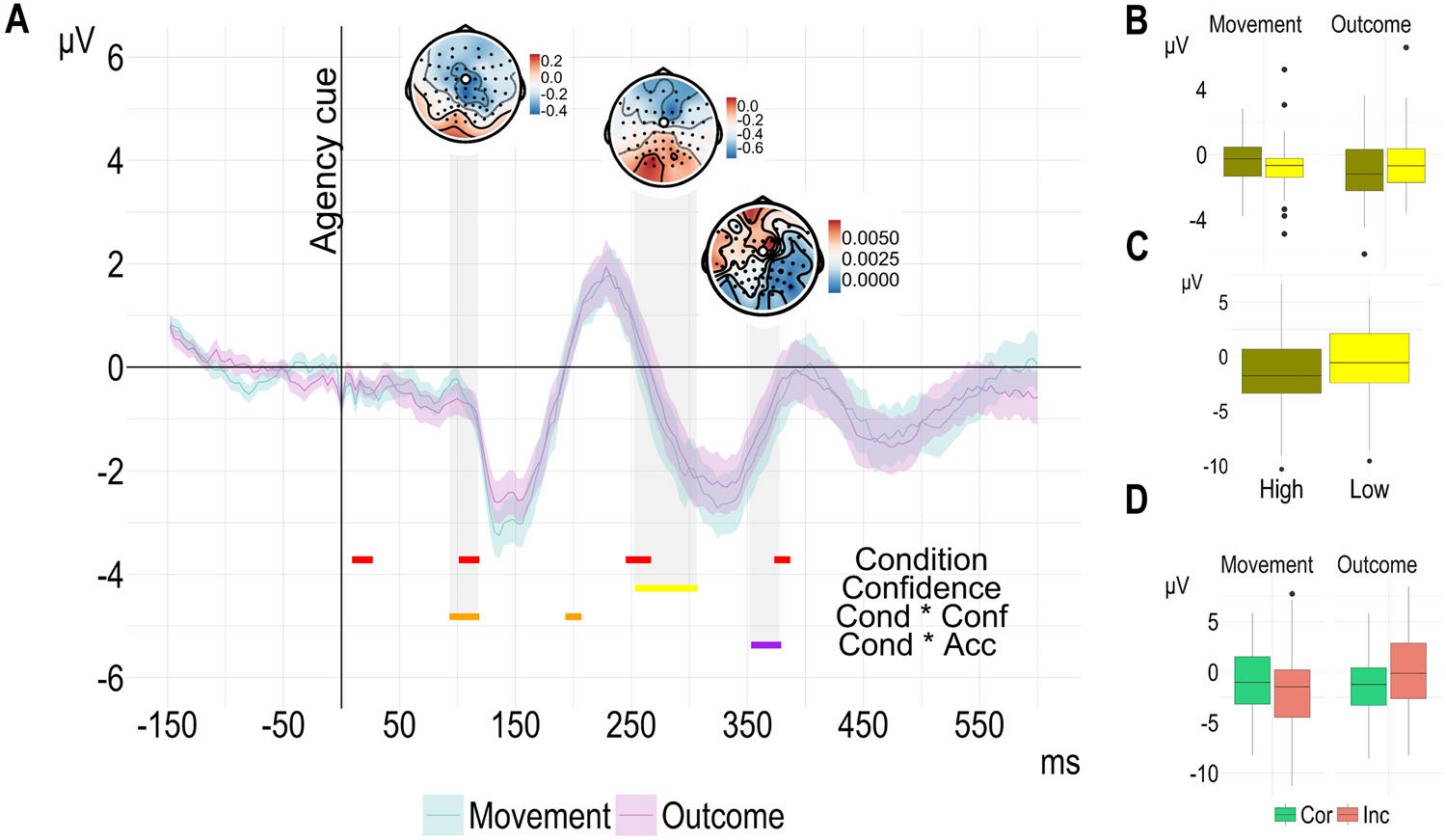
EEG amplitude for the two conditions (FCz electrode). ***A***, Time series: the signal was time locked to the onset of the discrimination cue (when participants provide their agency judgment). For illustration purposes, in this figure we averaged the epoched signal per participant and condition and plotted the mean of the data from the 37 participants included in this analysis. The ribbons around the mean represent the 95% confidence interval. The significant time point clusters from the cluster-based permutation analysis [EEG amplitude difference ∼ Condition * Accuracy * Confidence + (1 | participant)] are color-coded to indicate the effect they represent, marked on the bottom-right. The topographical maps show the mean beta estimates across the time points included in the clusters of the EEG amplitude marked within the gray area for each of the 62 channels recorded. The boxplots show the mean EEG amplitude across the time points included in the clusters (areas marked with gray) linked with the interaction between condition and confidence (***B***), the main effect of confidence (***C***), the interaction between condition, and accuracy (***D***). To simplify the visualization, we median split the continuous confidence ratings for each participant and used the resulting binary factor of confidence (“High” vs “Low”) only for this figure.

When we explored differences in the epochs time locked to the discrimination cue, we found four significant clusters linked to the effect of condition (onset ∼12, 104, 248 and 372 ms); one cluster linked to confidence (starting ∼256 ms); one cluster linked to the interaction between condition and discrimination accuracy (onset ∼356 ms); and one cluster based on the interaction between condition and confidence (onset ∼96 ms). As shown in Figure 5, by averaging the signal of all the time points included in the confidence cluster, we found that high confidence was linked with smaller EEG amplitude compared with low confidence. However, when averaging the signal of all the time points included in the cluster associated with the interaction between condition and confidence, we noticed that the amplitude of the EEG signal was greater for high-confidence Movement trials compared with low-confidence Movement trials and smaller for high-confidence Outcome trials compared with low-confidence Outcome trials (onset ∼96 ms). This result suggests that the confidence level is linked with the amplitude of the EEG signal already when participants are prompted to provide their discrimination response and varies by the type of feedback manipulation introduced during the action. Similarly, by averaging the signal of all the time points included in the cluster associated with the interaction between condition and discrimination accuracy (onset ∼356 ms), we see that the amplitude of the EEG signal was greater for correct Movement trials compared with incorrect Movement trials and greater for incorrect Outcome trials compared with correct Outcome trials. This result suggests that the neural signal differs between conditions during the Discrimination cue epoch and implies the presence of different underlying mechanisms engaged based on the type of agency representation being manipulated.

(B) Confidence response

The permutation analysis on the epoched data time locked to Confidence cue revealed five significant clusters. The first was linked to the effect of condition (onset ∼24 ms), the second to the effect of discrimination accuracy (onset ∼36 ms), the third and fourth to the effect of confidence (onset ∼136 ms; ∼216 ms), and the fifth cluster indicated an interaction between confidence and discrimination accuracy (onset ∼40 ms). The time points included in the cluster indicating an interaction between confidence and discrimination accuracy overlap with those associated with the main effect of discrimination accuracy. Therefore, we focus here on the interaction effect. By averaging the signal across all time points within the cluster associated with the two-way interaction between discrimination accuracy and confidence, we found that the amplitude of the EEG signal was greater when participants correctly identified the interval in which they were in control compared with when their decision was incorrect. This difference was more pronounced for the low-confidence trials compared with high-confidence trials. Overall, though, low-confidence trials were associated with smaller EEG amplitude compared with high-confidence trials, as evidenced by averaging the signal of all the time points included in the second confidence cluster (onset ∼216 ms). Finally, by averaging the signal over the time points included in the condition cluster, we found that EEG signal amplitude was greater in Outcome trials than that in Movement trials. Taken together, the results from the response cues suggest, first, that violations of movement representation prediction are associated with distinct neural processes compared with violations of outcome representations when participants assess their agency through explicit agency reports and then rate their confidence in their decisions and second, that both response accuracy and confidence levels can predict the neural signal during these assessments.

## Discussion

We tested the role of movement and outcome information in metacognitive representations of agency. That is, we measured the clarity of the subjective experience of agency when either the visual feedback of a movement (Movement condition) or its proximal outcome (Outcome condition) is manipulated. Participants performed a goal-oriented action two consecutive times. Then, they discriminated on which of the two actions they felt more in control and rated their confidence in that decision. Our study showed that Mratios of the Movement and Outcome conditions did not differ but they also did not correlate. We also found evidence for distinct EEG signals when we prompted participants to register their agency (discrimination) and confidence reports. Here, we will argue that, overall, the results of the behavioral and EEG data are in line with the idea (Kalckert and Ehrsson, 2012; Christensen and Grünbaum, 2017; Wen, 2019) that bodily and external agency should not be collapsed into a single construct. In what follows, we discuss each of these pieces of evidence separately and then bring them together to discuss how they might inform more theoretical accounts of agency. We will also, however, highlight the limitations of this study and encourage further work that might provide stronger evidence for or against a neurophysiological distinction of the two conceptually distinct kinds of agency.

The absence of differences in Mratio between the two kinds of manipulations suggests that metacognitive representations of agency are equally sensitive to movement and outcome prediction violations. This result goes against our preregistered hypothesis that the mean Mratio would differ between the two conditions. Instead, we found in fact evidence for the null hypothesis of no differences between the conditions. Hence, this result suggests that the salience, or precision, of experiencing a violation of predictions following an action is not consistently different, for the sampled population, depending on whether those predictions are bodily or external. Because we measured the precision of metacognitive representations, and not, like previous literature, merely mean agency ratings, a direct comparison between these results and previous studies must be taken with caution. These two representational levels—agency and metacognitive judgments about it—have been argued to be distinct, both behaviorally (Charalampaki et al., 2024) and computationally (Constant et al., 2022). Nevertheless, these results do complement the field. Previous studies examining the relative importance of movement and outcome prediction violations in participants’ sense of agency have yielded conflicting findings. Some studies have argued that participants’ sense of agency is more sensitive to outcome violations than to movement violations (Wen et al., 2015; David et al., 2016; Oishi et al., 2018). Other studies have provided evidence for the opposite, namely, that participants’ sense of agency is more sensitive to movement violations relative to outcome violations (Metcalfe and Greene, 2007; Metcalfe et al., 2013).

In addition to using metacognitive judgments of agency, our study differs from previous work in two important ways: (1) the use of qualitatively comparable feedback manipulations in the two conditions and (2) an experimental design in which the distal goal was reached on almost every trial for all participants. Regarding the first point, in much of previous work, the manipulations compared are typically qualitatively different from one another. For instance, in movement manipulations, visual feedback is usually continuous and presented for a sustained period of time; whereas in outcome manipulations, visual feedback is usually discrete, presented for a shorter duration, and typically consists of a sound or color change at the end of the action (Wen et al., 2015; David et al., 2016; Oishi et al., 2018) or the outcome congruency (Metcalfe and Greene, 2007; Metcalfe et al., 2013). This comparison is problematic because previous work has shown that the duration of the feedback can overshadow or conceal the true effect of movement and outcome prediction violations. Schmitter et al. (2021) found that continuous visual feedback differed from discrete feedback not only in participants’ accuracy in detecting a delay but also in the BOLD activity patterns. The authors interpreted these results as indicating that distinct predictive mechanisms underlie the processing of the two different types of manipulation (Schmitter et al., 2021). In our study, we manipulated the visual feedback of both the movement and the proximal outcome continuously. Put simply, we manipulated either the visual representation of participants’ hand movement or the ball flight trajectory, both of which have a long duration relative to the short-lived outcome representations employed in previous studies. Therefore, we argue that the Skittles task allowed us to compare the two types of action representations in a tightly controlled manner, minimizing response and comparison biases. This does not undermine the findings from previous studies but rather highlights the limitations of comparing simple subjective reports between experimental conditions that are known to participants.

Regarding the distal goal, Kumar and Srinivasan (2014) showed how goal-dependent the relative role of movement and distal outcome information is: In a multi-agent task, they found that when participants successfully achieved their goal, they rated agency independently of their motor performance. However, when participants missed their goal, they relied more on the sensorimotor information (Did what I did match what I saw?) rather than the distal outcome congruency to rate their agency. Further, van der Weiden et al. (2010) showed that there are interindividual differences in the way participants represent their behavior (movement vs distal outcome) and that these representations can be prompted by the use of subliminal primes (prior to the action) or specific instructions (“make a specific movement” vs “reach a specific goal”). Together, these studies suggest, first, that reaching a goal can affect agency ratings following outcome manipulations differently than ratings following movement manipulations and, second, that different participants can differ in these relative effects. Importantly, we designed the virtual Skittles scene in such a way as to ensure participants would be successful in meeting their goal of hitting the target in the majority of the trials. On rare occasions, when participants missed the target in either of the intervals, we excluded the trials from further analysis. Hence, we argue that goal-based expectations were not a confound in our design.

Further, the second finding of our study was that participants’ Mratio following movement manipulations did not correlate with Mratio following proximal outcome manipulations. Simply put, those participants in our sample who were most metacognitively sensitive to movement violations were not always those most sensitive to proximal outcome violations. This result suggests that a simple comparison between mean judgments of agency following different kinds of manipulations might not be sufficient to reveal the dissociations between them: Two different manipulations might affect the experience of agency to the same extent and nevertheless be processed via distinct mechanisms or contribute with different weights to the overall experience of agency (partial independent mechanisms). Because our preregistered hypotheses focused on the mean differences between conditions, we determined our sample size based on power analyses to detect mean differences rather than correlations; hence, we remain cautious with this interpretation.

Finally, when examining the EEG signal recorded at FCz during the time of the discrimination and confidence decisions, we found it to reflect the accuracy and confidence of the decision along with the type of violation prediction. At this point of the trial, participants are discriminating between two action epochs; thus, what we measure here is the underlying signal of the agency salience comparison between those two preceding action epochs. The association of the amplitude difference with the type of violation prediction suggests that the brain can dissociate the two types of violation based on the salience of the experience they affect. Together, the effects of condition at the time of the discrimination and confidence decision suggest that the salience of the agency experience might vary between kinds of manipulation. Further work should test this potential dissociation by extending our analysis to other cortical regions linked to the sense of agency and employing advanced methods (e.g., connectivity analysis, multivariate decoding) to better characterize the underlying signal of each.

### Bodily and external representations and the underlying model of agency

To reiterate, our study showed that Mratios of the Movement and Outcome conditions did not differ but they also did not correlate. We also found evidence for distinct EEG signals when we prompted participants to register their agency (discrimination) and confidence reports. These results emphasize the need to not conflate bodily and external agency into a single unified experience. The comparator model (Frith et al., 2000) is often used to explain the experience of (or reduction in) both the bodily and external sense of agency. There exists evidence to support the involvement of a comparator mechanism in the bodily sense of agency (but see Christensen and Grünbaum, 2018). However, the involvement of forward models in the comparison between predicted and perceived sensory consequences that go beyond the body has been challenged (Christensen and Grünbaum, 2018; Dogge et al., 2019). While a previous study has shown that participants can predict the distal outcome of their actions in the environment before even receiving explicit feedback about it (Maurer et al., 2022), it cannot be automatically assumed that a copy of motor commands is responsible for the predictions about sensory consequences that go beyond the body (Christensen and Grünbaum, 2018; Dogge et al., 2019). The comparator could explain the role of motor incongruencies and, by extension, bodily representation violations in a diminished sense of agency. If there was evidence that the comparator model could make predictions based on the forward models for sensory information external to the body, it would mean that the comparator could explain the role of outcome incongruencies and, by extension, violations of bodily and external predictions should result in both correlated metacognitive agency representations and indistinguishable underlying signal when participants are prompted to report their discrimination agency decision. However, based on our results, it seems less likely that a single comparator mechanism is responsible for processing violations of both representations. While a formal model comparison is beyond the scope of this work, we speculate that a more inclusive model of agency that extends the core ideas put forward by the comparator model—like the Bayesian cue integration theory (Moore and Fletcher, 2012; Synofzik et al., 2013)—might better align with our results. The Bayesian cue integration theory, for example, posits that a sense of agency results from a weighted combination of different cues and puts forward a computational mechanism to integrate these cues, some of which result from a comparison similar to that supported by the simplest form of the comparator model, and other cues that may not. The weighting assigned to each of the cues can be determined by priors (prior knowledge and expectations). Because the model is, first, agnostic about how exactly these sources of information arise and, second, because it suggests that these cues are integrated, the cue integration model may be more suited to account for our results, specifically the differences found due to the salience of the agency experience. However, this claim remains speculative at this point and requires further validation. For instance, to conclusively argue for the distinct contributions of movement and outcome prediction violations, future studies might operationalize both types of violations within the same trial. Specifically, introduce violations of bodily representations in one of the intervals and violations of the external representations in the other, while varying the magnitude of manipulation for each one used within the trial. Then, as we did here, test for differences in participants’ metacognitive representations of agency, behaviorally and at the neuronal level. This design would allow one to test if the absence of correlation we found in our study and the distinct brain signal is the result of a weighted contribution of different sources of information while forming representations of agency and run computational models to identify the way the brain assigns different weights to each agency representation.

### Limitations

In this study, we used Mratio to compare the precision of two potentially distinct agency representations: bodily and external. This metric allows for a direct comparison of the two otherwise not-comparable representations, but it may be influenced by first-order discrimination performance. To ensure this was not the case in our study, we implemented separate staircase procedures. This approach enabled us to examine how two types of prediction violations (movement related and proximal outcome related) influence metacognitive representations of agency, beyond mere differences in discrimination performance (but see Guggenmos, 2021; Rausch et al., 2023). However, as Rahnev and Fleming (2019) have noted, using staircases carries the risk of inflating estimates of metacognitive efficiency. Beyond this, we observed high variability in Mratio, which may be driven by factors such as stimulus strength and variability. Although we accounted for both the magnitude of the manipulation and staircase variability (used here as a proxy for stimulus variability), in our analyses of confidence ratings and Mratio, this remains an inherent limitation of our design. More broadly, when comparing the influence of bodily versus external information on the formation of agency representations, we are necessarily comparing two components of action that differ substantially in both space and time. Until more refined methods for assessing metacognitive abilities are available, we argue that our approach provided a suitable framework for addressing these questions.

Finally, we investigated the role of bodily predictions’ violations by manipulating the visual representation of participants’ hands, which appeared on the screen as a simple rectangle. Previous research has shown that participants can experience ownership over a variety of visual representations of their body parts (Kondo et al., 2018). Thus, we consider our results relevant to understanding the sense of bodily agency. Nevertheless, future work should aim to replicate and extend our findings using more realistic visual representations of bodily movements.

### Future directions

We used a metacognitive task to study the subjective experience of agency devoid of response biases. Another option would be to study agency with implicit measures. For example, temporal binding consists of biases in the perceived timing of the movement and the outcome of voluntarily performed actions (Haggard et al., 2002): movement initiation is perceived as happening later, and outcome as happening earlier, than they really did. Alternatively, sensory attenuation, where the sensory consequences of voluntarily performed actions are perceived as less intense (Blakemore et al., 2000) compared with those of passive movements, is also sometimes used to quantify the sense of agency. However, these measures do not directly address the subjective component of agency; rather, they act as a proxy. In particular, there is an ongoing discussion on the link between implicit and explicit measures of agency, the details of which are outside the scope of this manuscript. Briefly, the discussion revolves around whether these two kinds of measures target the same phenomenal experience. While some studies argue that they do (Imaizumi and Tanno, 2019), others argue against it (Dewey and Knoblich, 2014; Schwarz et al., 2019). Therefore, it would be interesting to compare the role of bodily and external representations on agency with implicit agency measures. Not only to inform the discussion of implicit versus explicit measures, but also to compare our results using a metacognitive measure and those from studies using implicit measures. Given the nature of our design, where participants perform an action and receive visual feedback that is spatially incongruent with their action (either at the level of the movement or the level of the proximal outcome) adapting it directly into an implicit agency task may be challenging. However, an alternative motor task can be developed to clearly distinguish between the bodily and external aspects of agency allowing adjustments suitable for both metacognitive and implicit agency designs.

### Conclusions

We found that the sensory information of both the movement and the outcome is important for experiencing being the author of an action we intentionally perform. However, we should not treat these two sources of information as interchangeable as this overlooks a possible distinct contribution to an overall sense of agency as suggested by our results. Further research is needed to understand how movement and outcome representations inform our sense of agency.

## Synthesis

Reviewing Editor: Frederike Beyer, Queen Mary University of London Decisions are customarily a result of the Reviewing Editor and the peer reviewers coming together and discussing their recommendations until a consensus is reached. When revisions are invited, a fact-based synthesis statement explaining their decision and outlining what is needed to prepare a revision will be listed below. The following reviewer(s) agreed to reveal their identity: Wen Wen, Nura Sidarus.

I have received detailed comments on your manuscript from two experts in the field. As you can see, both highlight the potential novelty and merit of your work. However, both also raise important issues around the analysis and interpretation of data, in particular the EEG data. Upon reading the manuscript myself, I agree with the comments of both reviewers, that in its current form, the analysis approach for the EEG data lacks a clear rationale, and as such the results are difficult to interpret. In your revision of the manuscript, please consider the suggestions made below. Reviewer 1 suggests frequency-domain analysis may be more appropriate for your paradigm, and as both reviewers highlight, the current analysis approach would benefit form providing clear hypotheses. Where differences between amplitudes are interpreted as evidence for differences in sense of agency, this should be supported by relevant literature, which should be discussed in the introduction. If the EEG analysis is entirely exploratory, this needs to be clarified, and the interpretation of results adjusted accordingly.

Depending on which additional analyses you may choose to conduct and in how far an evidence-based approach can be taken, I would then encourage you to consider which results are best presented and discussed in the main manuscript, and whether any might be better moved to the appendix, to allow for a clear presentation and discussion of findings.

Both reviewers also highlight important points regarding the interpretation of behavioural data, which you should address in your revision.

Reviewer 1

The authors examined the sense of agency over movement and outcome using a metacognitive framework. They found that the M-ratio did not correlate between the two conditions in which either the movement feedback or the outcome feedback deviated from the actual events, although the M-ratio values did not significantly differ between these conditions. The authors also measured EEG signals during the task and found that condition, confidence, and accuracy had different effects on the amplitude difference between the actual feedback condition and the disturbed condition during the movement and outcome epochs. Based on these findings, the authors concluded that the experience of agency over movements and outcomes is governed by different mechanisms.

First, I believe the use of a metacognitive framework has its merits. It is generally difficult to directly compare the sense of agency across conditions in which either movement or outcome is violated, as task difficulty often differs. The use of an online staircase procedure allows control over first-order task difficulty, and the M-ratio serves as a useful index of how well participants can access their agency judgments. However, this method also has limitations. Notably, a difference in M-ratios alone does not necessarily imply different underlying mechanisms, as it simply reflects differences in metacognitive efficiency under varying feedback conditions.

Moreover, the source of the large individual differences in metacognitive efficiency remains unclear, making it challenging to draw reliable conclusions from observed correlations-or the lack thereof-between individuals. In this study, the primary behavioral result supporting the conclusion is the absence of a correlation between M-ratios in the movement and outcome violation conditions. Figure 3 shows that, although individual variability in first-order accuracy was small due to the staircase procedure, variability in M-ratio was large. There is no clear explanation for this variability, making it difficult to interpret whether a lack of correlation truly indicates distinct mechanisms.

In addition, the authors did not discuss the first-order task performance in detail. While d' was controlled across participants, the actual task difficulty (e.g., the velocity manipulation) likely varied. Did the staircase velocities correlate between the movement and outcome violation conditions? If so, individuals who are better at detecting movement violations might also excel at detecting outcome violations. If not, this would suggest that sensitivity to these two types of violations differs within individuals, which would support the authors' hypothesis. This result should be reported.

Furthermore, the conclusions drawn from the EEG analysis are unconvincing. The authors analyzed the amplitude differences between the violated and non-violated conditions using a linear mixed-effects model to examine the influence of condition, confidence, and accuracy. However, the rationale for using amplitude differences as an index of agency-related processing is unclear. For example, even if confidence influences EEG amplitudes during movement epochs but not outcome epochs, it remains difficult to interpret what cognitive processes are being reflected. EEG signals recorded during movement are also susceptible to artifacts, and movement could be confounded with task performance, potentially leading to spurious correlations between subjective judgment and EEG amplitude. I do not believe amplitude-based analysis is suitable for a task involving overt movement. Additionally, the high rejection rate of epochs due to artifacts raises concerns about the robustness of the findings. The authors should justify their choice of amplitude analysis over alternative approaches, such as frequency-domain analysis.

In summary, the authors' discussion of whether the sense of agency over bodily movements and over subsequent outcomes involves different mechanisms remains unclear. If the mechanisms do differ, in what way? Merely showing differences in sensitivity or metacognitive efficiency between the two conditions is not sufficient, as such results may only reflect differences in the metacognitive accessibility of agency-related information. The absence of a significant correlation in M-ratios across individuals does not provide strong evidence for distinct mechanisms. Moreover, the EEG analysis lacks theoretical justification and is insufficient to support the authors' conclusions given the ambiguity surrounding what the amplitude differences actually represent.

Finally, it is difficult to envision how the "skittle ball" behaves or how strongly participants might experience a sense of agency in this task based on the figures alone. It would be helpful for readers if the authors could provide a video demonstration of the task, showing the movement of the virtual lever and the trajectory of the flying ball.

Reviewer 2

The authors present an interesting study and approach for studying the metacognition of agency. Using a task that allows for comparable manipulations of the visual feedback of one's bodily movements and their external consequences, both with continuous feedback, extends previous research on the relative impact of movement vs outcome related cues on agency. The behavioural results demonstrate no significant differences across the two cues in the various measures of behavioural and metacognitive performance, suggesting both may be monitored independently, rather than one mattering more than the other. This is a relevant contribution to the literature, given the inconsistencies in previous findings. Nonetheless, there are some issues that warrant revisions related to the interpretation of results, the EEG data, and more minor editing issues.

1. It's worth noting that Rahnev &Fleming 2019 https://academic.oup.com/nc/article/2019/1/niz009/5513064 have shown that metacognition measures (as used here) are altered by using a staircase procedure during the trials (relative to fixed). This could inflate estimates, and such inflation is related to stimulus variability. Therefore, it would be worth discussing the potential impact of that design choice to the results describe here (in the limitations), as well as assess whether stimulus variability may differ between the two conditions.

2. P. 30 - "One might wonder if goal-based expectations could explain this dissociation. Previous studies would support this interpretation." - I don't follow the logic of this argument, or what the authors are trying to get at, so would be best to rephrase. Just before, the authors argue that their results suggest that different disruptions of agency may affect it to similar extents (in terms of ratings / metacognitive reports), but through different mechanisms (which I agree with). But I didn't understand why goal-based expectations would be relevant to explaining this effect? Or how the studies that follow would support thinking that goal achievement is relevant here (since it was always achieved). Indeed, a difference in the mechanisms for (or distinctness of contribution for) monitoring movement vs outcome related information seems required by the different nature of the cues involved (with studies having also shown different neural mechanisms). Related, I also do not follow the argument of the ensuing paragraph ("To conclusive argue..."), which seems to just be arguing for a replication of their study, but doesn't clarify what type of evidence they are arguing could demonstrate there being a "distinct contribution" vs demonstrate no distinctiveness? Overall, it's unclear to me what's the issue/question at stake here.

3. P. 32 - the discussion of the comparator model here perhaps relates to issue above. But this seems like a straw man argument, since the comparator model is a highly abstracted, rather than truly mechanistic model. This seems to argue against the equivalency of processes that cannot mechanistically be identical, since different signals would be compared anyways across the different levels of monitoring of the hierarchies of movement vs proximal vs distal outcome. The logic of the comparator model is aligned with the logic of predictive coding models (also highlighting those hierarchies in predictions), and it's unclear what would be the basis for deriving an outcome prediction (for detecting violations) if it wasn't from information about the action/movement performed? Plus, when claiming the comparator model logic isn't supported by "our results", they should clarify which exactly they mean, since the results do support a similar impact of either deviation signals on agency.

4. No clear justification is provided for the inclusion of EEG in the study, and no EEG related results are reviewed in the introduction. While understandable that this may have been a largely exploratory part of the study, it seems surprising to have invested such effort with no hypothesis at all. Such a lack of grounding on any past findings linking EEG signals to action or outcome monitoring, or agency, renders it very difficult to understand how the EEG analysis could be approached and how any results could be interpreted. From the discussion, it seems a possible purpose for this was to support claims about potential differences in neural mechanisms involved in movement vs outcome monitoring, and hence in the mechanisms underlying respective violation detection, independently of the magnitude of effects on explicit reports. In which case that logic should be clarified the introduction as the motivation for including such methods (though also seem worth acknowledging the issues underlying that logic raised above).

5. EEG Data analysis:

a. It is very difficult to interpret the results, given the lack of any hypothesis-driven selection of relevant data dimensions to consider. Moreover, having computed a difference score, it seems problematic to interpret the resulting effects on the "difference score" in a linear way (i.e. that more a positive value means a larger difference between the amplitude observed when feedback was manipulated or not), given that any deviation away from 0, whether positive or negative, would effectively index a "larger difference". 3-way interactions are inherently difficult interpret, and it's unclear what is the statistical grounding for interpreting any of the pairwise comparisons that are described (and it's also confusing what's meant by "larger difference" and whether that's an appropriate interpretation given the point above). E.g. in p. 21, where the authors created Fig. 5.B to probe the 3-way interaction effect, and then offer some statements describing some pair-wise effects (later also in p. 23 of the outcome epoch). However, at the figures (5 and 6 B), it seems very unclear what's the statistical basis for those descriptions given the large overlap in the confidence intervals and similarity in the means. Notably, in the context of a significant interaction, the main effects can't be interpreted alone. Although this would constitute double dipping, thus inflating effects, given the stated exploratory context of these analyses, it wonder if it might be more revealing to run a new specific mixed effect model that matches the figure(s), using the average signal in the selected time window. In which case, the full model results for that analysis could be clearly reported, and they could further explore the interactions by looking at the model predictions (e.g. with sjPlot and emmeans in R), to more clearly visualise and get better sense of whether/where there are robust differences in the effects.

b. The descriptions of the results in the discussion are also confusing (ensuing subpoints), and indeed I found myself confused about the effects depicted on e.g. Fig.5, namely regarding whether the differently coloured traces are effectively representing the condition effect? As it then seems quite surprising that there are many cases (across figures) of seeing large differences between the two depicted traces, with no overlap in confidence intervals, and yet no significant effects picked up by the cluster-based permutation analysis?

i. P. 30 - "First, by comparing epochs while the..." - Please rephrase, to clearly remind the reader what they mean by "comparing" here (i.e. relative to what?), which is important also when later describing the "second" set of analysis of epochs around judgements, as the "first" analysed a difference score, and the "second" set just the raw EEG signal. The analysis of the confidence judgments is also missing within that sentence describing the "second" set of analyses, as well as in the discussion section overall.

ii. Related, P.30 - "a direct comparison between EEG epochs is also granted, although they corresponded to different parts of the epoch" (p. 30) - please rephrase as the repeated use of "epoch" to mean different things gets quite confusing, and it's again unclear what "comparison" is being referred to? As it can seem to imply potential comparisons between the activity during the movement (outcome) phase of a trial, rather than the manipulation conditions.

iii. In fact, across sections (but e.g. also in the next paragraph), its gets quite confusing that sometimes the same words - "movement" and "outcome" - are used for describing a phase of a trial vs. the condition and manipulation type. Please review for consistency, e.g. by ensuring that an extra qualifier (e.g. movement "epoch" vs "condition") are consistently used, to minimise confusion.

1. E.g. p. 31 - "However, only during movement manipulation did the EEG signal recorded reflect this relationship for a sustained interval (Figure 5). In contrast, the EEG signal recorded during proximal outcome manipulations did not vary with participants' confidence, or with their discrimination accuracy (Figure 5)." - it's unclear what is the evidence supporting those claims? If specifically referring to the "movement epoch", the 3-way interaction effect seems too confusing to so conclusively interpret in that way (plus the following statements). Plus, this would seem to draw a general conclusion about EEG activity across the whole trial, when there was no discussion of specific effects observed during the "outcome epoch". In fact, the allusion to a "sustained" effects seems rather to point to contrasting here the longer window of some interaction effects observed during the "movement epoch" relative to during the "outcome epoch", rather than during one or the other condition. It's then also unclear how the ensuing interpretations about the timing of when different predictions violations are detected holds (e.g. if in the "outcome epoch" there was also some 3-way interaction effect, very shortly after the ball became visible).

c. When describing the linear mixed effects models, please include a clear description of how predictors were coded in the model (e.g. dummy coding? which is the baseline?). In fact, using contrast/sum coding of the binary predictors (e.g. 0.5/-0.5) could greatly facilitate model estimation and interpretation (perhaps even allowing continuous confidence ratings), as it ensures that all predictors are estimated relative to the average across the other predictors (rather than relative to the baseline condition in a dummy coded predictor). While I understand that the full cluster-based permutation analysis may too computationally expensive to redo, it may be worth considering for the analysis suggested above.

d. Related, it seemed very strange to argue that "we prefer not to interpret the timing of the clusters" p.17, when that seems like the only information that could be glimpsed from such analyses. Indeed, this claim is undermined by later referring to the timings of effects in the discussion ("already within 250 ms after the manipulations took place" p.31). Therefore, the statement in the methods section should be reviewed, with further clarity added on what they would be intending to learn from such analysis.

6. The discussion of the possible use of implicit measures (p. 33) does not seem very relevant to this specific study, or future directions, since it's unclear how such methods could possibly be combined with the type of design proposed here, to allow comparisons with such metacognitive measures? Or at least that should be acknowledged, or better explained.

7. Regarding the task design: The "Movement condition" is described as: "the virtual lever always moved synchronously with the physical one but had a different angle, and the ball flew according to the participant's actual throw (based on the angle of participants' real hand and velocity at the point of ball" (p.8). - This implied a potential disjunction/jump in the ball location observed - as also implied visually in Fig 2.B. Please make clear also in the methods section and figure, what is later described in p.22, about how the ball was hidden for 250 ms after release prevent that (as it wasn't obvious then).

8. Data exclusions: it is noted that "the first four blocks from two participants" (p.12) were excluded, which is almost half of their data, hence it seems surprising that the remaining data could be enough to still include? Relatedly, though the authors thankfully summarise somewhere the final mean and SD of the trials included across the subsets of analysis, it would also help to see the minimum number included, to get a better sense of the variability and of the possible "worse case" scenarios.

9. p.3 - When mentioning research that does not follow the first pattern described that outcomes matter more than means for SoA, only two studies involving the same task are cited, whereas many were cited to support the first pattern. Even if not explaining in much detail, for conciseness, it seems worth including references to other studies/designs that don't align with the first pattern, e.g. Caspar et al 2015 http://www.sciencedirect.com/science/article/pii/S1053810015000082 showing that violations of sensorimotor cues can abolish the effect of outcome violations; or the Villa et al studies later cited in the discussion. Notably, Villa et al 2018 is grouped with studies showing that sensorimotor cues matter more than outcome cues, but that studied showed no robust interaction effect, thus seeming more aligned with an interpretation where both cues matter.

10. A link to the data and code was included and indeed that link includes those components. Yet, I note that a pre-registration was mentioned, but no link to it was identified to verify it (and it didn't include that). If that was to maintain anonymity, I trust the editors will review that.
